# Moving from trust to trustworthiness: Experiences of public engagement in the Scottish Health Informatics Programme

**DOI:** 10.1093/scipol/scv075

**Published:** 2016-05-11

**Authors:** Mhairi Aitken, Sarah Cunningham-Burley, Claudia Pagliari

**Affiliations:** ^1^Centre for Population Health Sciences, Medical School, University of Edinburgh, Teviot Place, Edinburgh, EH8 9AG, UK

**Keywords:** public engagement, trust, health informatics, data linkage.

## Abstract

The Scottish Health Informatics Programme (SHIP) was a Scotland-wide research programme exploring ways of collecting, managing and analysing electronic patient records for health research. As part of the SHIP public engagement work stream, a series of eight focus groups and a stakeholder workshop were conducted to explore perceptions of the role, relevance and functions of trust (or trustworthiness) in relation to research practices. The findings demonstrate that the public’s relationships of trust and/or mistrust in science and research are not straightforward. This paper aims to move beyond simple descriptions of whether publics trust researchers, or in whom members of the public place their trust, and to explore more fully the bases of public trust/mistrust in science, what trust implies and equally what it means for research/researchers to be trustworthy. This has important implications for public engagement in interdisciplinary projects.

## 1. Introduction

Public trust in science is a subject of much academic and policy discussion with many international bodies seeking to ‘improve’ public trust in order to address a perceived threat to the public authority of science (Bates et al. 2010; Wynne 2006). At least since the UK House of Lords Science and Technology Committee’s landmark statement that there was a ‘crisis of trust in science’ (House of Lords Science and Technology Committee 2000), it has routinely been suggested that a series of high-profile scientific controversies and scandals (e.g. BSE, thalidomide and the MMR triple vaccine), together with the rapid pace of scientific progress have resulted in an erosion of public trust in science. This is considered significant since:

… science and technology demand assenting publics to maintain their hold on the collective imagination, not to mention the purse-strings. (Jasanoff 2005: 248)

As such, considerable attention has been paid to ‘improving’ public trust in science. Most notably, this has been pursued through efforts to increase public understanding of science, on the assumption that, where the public is sceptical or mistrusting, this can be explained by ignorance or lack of understanding, and as such can be ‘corrected’ through better dissemination of scientific knowledge or ‘facts’ (Jasanoff 2005; Wakeford 2010).

Public understanding of science (PUS) explanations of public mistrust have come to be widely criticised and discredited on a number of grounds. First, they present the public as ‘passive recipients of scientific knowledge’ (Cunningham-Burley 2006: 206) and imply that science inevitably has the ‘right’ answers (Yearley 2005). Secondly, the underlying assumption that greater knowledge or understanding of science results in greater acceptance remains unproven. Understanding is not a simple process through which individuals simply or straightforwardly receive the ‘correct’ knowledge but rather ‘lay’ publics actively deconstruct, question and evaluate claims to scientific knowledge (Hagendijk and Irwin 2006). Thirdly, PUS has been criticised for its lack of reflexivity. While it has sought to explain a perceived lack of public trust in science, it has overlooked considerations of what it means for science to be trustworthy (Wynne 2006). As Wynne (2008: 21) contends:

We cannot properly conduct relevant research on publics in relation to science, unless we also critically examine the elephant in the room - what is the ‘science’ which we are supposing that people experience and sense in each of these situations?

For these and other reasons, since the turn of the century there has been a move away from top-down efforts at ‘improving’ public trust through education or awareness raising and a:

… general recognition of the need for two-way dialogue between the public, on the one hand, and scientists and policy-makers on the other. (Wakeford 2010: 87)

Public engagement with science has now largely replaced PUS as the key mechanism for addressing the ‘crisis of public trust’ (Wynne 2006). Yet the underlying justification remains unchanged and the goal continues to be ‘improving’ public trust in science. Thus, such approaches remain largely unreflexive and overlook important considerations of what it means for science to be trustworthy, or what institutional arrangements lead to public trust/mistrust/distrust (Wynne 2006). As Wynne (2006) contends, while PUS suggested a deficit of public understanding, public engagement with science has replaced this with a deficit of public trust to be addressed through more information or more transparency. This remains a top-down approach and important considerations of institutional arrangements, or of what the publics are mistrustful of, are overlooked.

Moreover, the presumption that a ‘crisis of trust’ has come about as a result of a series of controversies or scandals which rocked public confidence in science is largely unquestioned. However, Wynne (2006) suggests that there may never have been a time when the public unquestioningly trusted science. Rather, public trust in science has always been conditional and ambivalent. Indeed, there is much evidence to suggest that general public support for science coexists alongside ambivalence and scepticism (Haddow and Cunningham-Burley 2008; Cunningham-Burley 2006; Wynne 1996). As Wynne (2006: 212) notes:

… there is lots of enthusiasm for [science] – but this is discriminating enthusiasm.

The public’s relationship with science is too sophisticated to be characterised by a simple trust/distrust binary relationship. Rather in many cases publics adopt an ambivalent form of trust – described by Wynne (1992, 1996, 2006) as an:

… ‘as-if’ trust – in response to their ‘knowingly inevitable, and relentlessly growing, dependency upon expert institutions. (Wynne 2006: 212)

Given the central role of scientific knowledge within society, publics have little choice but to trust in science. But this trust remains conditional and does not mean that they will inevitably have confidence in the scientists or scientific institutions conducting research.

Public trust in science remains a valid subject of research but the aim should not be:

… to ‘fix’ the lack of automatic trust (of the public in science) that may concern scientific institutions. (Marks 2011: 544)

Rather, there is a need for more symmetrical and reflexive considerations of what it means for publics to trust science, and equally of what it means for science to be trustworthy. There continues to be a need to explore the relationships of public trust/mistrust in science in order to understand what this means for scientific research/researchers and for the position of science in society. In particular, research ought to reflect on what it means for research and/or researchers to be trustworthy and on what bases public trust is founded. This requires a more nuanced understanding of the nature of public trust and greater critical reflection on the institutional arrangements of science. To a certain extent this more nuanced approach is reflected in calls for responsible research and innovation (RRI) as highlighted by the European Commission’s Science in Society programme (Owen et al. 2012). There is no singular understanding of what it means for research and innovation to be responsible, though Owen et al (2012) have identified several core discourses in this area. However, there is a shift in emphasis from engendering public trust in science to ensuring that science is trustworthy. This shift has very significant implications for managing science–public relations.

## 2. The SHIP

Given such a shift, challenging questions are raised around the implications for research and researchers, particularly regarding how research can ensure public trust—or its own trustworthiness. This paper therefore reports on the findings of a public engagement project related to a large science programme and the ways in which trust was understood by both associated researchers and members of the public.

The SHIP was a Scotland-wide research programme exploring ways of collecting, managing and analysing electronic patient records (EPRs) for health research. SHIP researchers developed systems to work across institutional boundaries allowing both health and non-health data to be easily linked on a national scale while protecting patient confidentiality. This was intended to be a powerful tool for understanding patterns of health and disease in the population and for assessing the effectiveness of interventions in delivering public benefit (for more information see <www.scot-ship.ac.uk> accessed 20 Apr 2016). Given its ambitious nature, the project inevitably raised a range of social and ethical considerations (e.g. around the ways in which personal data are stored, accessed and used or relating to processes for safeguarding confidentiality and respecting individuals’ autonomy). A programme of public engagement activities was therefore included as one of the core projects within SHIP. This had a number of aims, including:

- To understand the Scottish public’s preferences, interests and concerns relating to the sharing of health data for research.
- To explore the extent to which the public supported SHIP’s aims.
- To ensure that SHIP operated transparently and in the public interest.

The initiation of SHIP reflects significant broad interest in secondary uses of health data (i.e. uses other than those for which they were initially collected). The Medical Research Council and Wellcome Trust (2006: 6) note that:

Recent years have brought many calls for the optimisation of data sharing for research, with the intention of deriving maximal societal benefit.

Following on from SHIP, this commitment to greater sharing of both health and non-health data in the UK has recently been reinforced by the launch of the UK-wide Farr Institute of Health Informatics Research and the Administrative Data Research Network. Internationally, there is also significant interest in potential uses of routinely collected data which has led to a number of programmes including the Western Australia Data Linkage System, Population Health Research Network (Australia) and Population Data BC (Canada).

With the expansion of research uses of data there has been a growing interest in public acceptability. In part, this relates to recognition of the importance of ensuring that data is shared and used in ways which are seen to be in line with public interests or preferences. The recent highly publicised controversy surrounding care.data in England has highlighted the importance of ensuring that the uses of the data are publicly accep*table*. Similarly the failed introduction of Australia’s National Electronic Health Record Systems (NEHRS) demonstrates the importance of fully engaging with and addressing public concerns, taking account of how such programmes reflect, or jar with public values (Garretty et al. 2014a,b). Thus, increasing attention is paid to the public acceptability of secondary uses of data and to ensuring that these uses are understood and supported by the wider public (from whom the data originate). Public acceptability—and public trust—is crucial for ensuring the legitimacy of current practices and systems of governance. As Bradwell and Gallagher (2007: 18–9) have suggested:

… personal information use needs to be far more democratic, open and transparent.

And this means:

… giving people the opportunity to negotiate how others use their personal information in the various and many contexts in which this happens.

Until recently the literature in this area has been dominated by practitioner perspectives and public views have been underrepresented or underreported. For example, Robling et al. (2004: 104) stated that:

The acceptability to patients of access to medical records without their consent has frequently been assumed. However, the lack of any evidence about the acceptability of such activities from the potential research subjects – members of the UK public – is striking.

Where studies have explored public attitudes towards secondary uses of their data they have typically focused around issues relating to the anonymisation of data or (lack of) consent mechanisms (Damschroder et al. 2007; Saxena et al. 2006; Trinidad et al. 2012; McGuire et al. 2008; Willison et al. 2003; Medical Research Council and Ipsos MORI 2007). Broader issues around how programmes such as SHIP are perceived and the extent to which they are trusted have, until now, received less consideration. However, the literature is increasingly pointing to the centrality of trust in shaping public attitudes and responses to the secondary uses of data (Damschroder et al. 2007; Davidson et al. 2013; Ipsos MORI 2014; Trinidad et al. 2010).

Early scoping work conducted by members of the public engagement team involved interviews with members of SHIP’s Scientific Management Group (SMG) to explore expectations and understandings of the role of public engagement within SHIP. These indicated that one of the key objectives that SMG members considered public engagement should aim to fulfil was ensuring public trust in SHIP. This highlighted the salience of public trust to research. However, among the members of the SMG there were subtle divergences between those who suggested that public engagement might increase public trust through increasing awareness and understandings (i.e. through information provision) and others who suggested that public engagement might provide insights which would allow them to adapt aspects of SHIP in order to reflect public preferences and/or address concerns and as a result ensure public trust. This is an important difference in perspective, which leads to different expectations of public engagement. It also reflects the different positions on trust outlined above. Some SMG members demonstrated a deficit model approach in suggesting that public engagement be used instrumentally to create public trust through awareness raising, whilst others advocated a more reflective approach and viewed public engagement as an opportunity to reflect public values, interests or concerns within SHIP—thus ensuring public trust through making SHIP trustworthy.

This illustrates some of the challenges encountered in conducting public engagement effectively within interdisciplinary projects. It also highlights a lack of clarity or consensus relating to the value or relevance of public trust and the public’s relationship with science. Thus, the interviews with SMG members raised important questions regarding the relationships of trust between publics and SHIP and of how SHIP might ensure high levels of trustworthiness. The growing body of literature on public attitudes to uses of data in health research has also drawn attention to the centrality of trust in shaping or informing public responses (Asai et al. 2002; Damschroder et al. 2007; Davidson et al. 2013; Ipsos MORI 2014; Trinidad et al. 2010). SHIP public engagement activities therefore sought to explore these issues further, and through deliberation with associated researchers and members of the public examined both existing relationships of trust and also opportunities for building trustworthiness into the design and operation of SHIP.

## 3. Methods

### 3.1 Focus groups

The first empirical stage of public engagement activities was a series of eight focus groups (conducted between October 2010 and February 2011) which explored public awareness of, and attitudes towards, uses and potential uses of EPR for health research. These involved a wide range of public groups across Scotland and a total of 50 participants from a diverse range of backgrounds. Participants were recruited through pre-established groups such as patient support groups (relating to diabetes and mental health), a youth centre (with both young people and youth workers), an organisation representing people from black and ethnic minority backgrounds, a group of nursing researchers and friendship groups from a variety of professional backgrounds (including law, social work and social science research). Focus group participants were sampled through purposive sampling focused on maximising diversity across the focus groups in order to access a broad range of viewpoints and perspectives. The aim was to have a diverse, rather than statistically representative, sample (Barbour 2007). It was important that individuals within each of the groups shared common traits or interests and, in most cases, were pre-acquainted as this meant that they felt comfor*table* and able to discuss the issues freely (Barbour 2007). In two of the focus groups one or more participant(s) were acquaintances of the researcher/moderator and acted as gatekeepers for recruiting other participants. As has been found in previous studies using focus groups (Munday 2006), this enabled a level of understanding of group dynamics and viewpoints which would not otherwise have been possible.

The first focus group was run as a pilot (with a group of social science researchers). However, given that this was successful in eliciting a range of valuable viewpoints and since little was changed in the topic guide after this pilot it was decided to include the findings from this focus group. The participants from the pilot focus group were all social science researchers and hence had an atypical awareness and understanding of research processes which they reflected on in discussing their personal experiences and their own position as individual subjects of data. Indeed, most focus groups included individuals with some experience of research (whether in a professional, voluntary or personal capacity). Including a wide range of perspectives, levels of experience and understanding within the focus groups is a strength of this study and enables reflection on the range of viewpoints expressed across diverse groups.

The groups took place across Scotland (in Edinburgh, Glasgow, North Lanarkshire, West Lothian, Aberdeen, Inverness and Moray) and included a diverse range of age groups (the youngest participants being 16 and the oldest in their 70s), a roughly even split of genders was achieved (with 27 female and 23 male participants).

A semi-structured approach was taken. A topic guide was developed to ensure a level of consistency between the focus groups. However, this was very flexible and allowed participants to raise issues and/or concerns which they considered to be relevant. The semi-structured design also meant that topics of discussion did not always arise in a pre-determined order, and that the focus groups were able to explore unanticipated areas of interest. As is recognised to be an advantage of focus group research, this approach allowed for a responsive, conversational style resulting in open and frank discussions and enabled individuals to engage with a topic which was previously unfamiliar to them (Barbour 2007).

### 3.2 Stakeholder workshop

As will be illustrated below, the focus group findings indicated, among other things, that trust was a highly salient factor influencing responses to SHIP. Given the relevance of trust in shaping public responses, it was felt that it would also be important to understand how trust is perceived and experienced by the range of actors who may use or benefit from SHIP (e.g. researchers, analysts, data controllers). Therefore, in collaboration with colleagues in the Information Governance work stream of SHIP, a workshop was held with a range of stakeholders during the SHIP biannual conference (9–11 September 2011). This explored stakeholders’ perceptions of the role, relevance and functions of trust (or trustworthiness) in relation to research practices. A total of 28 conference delegates participated in the workshop. The range of perspectives included: researchers, social scientists, government analysts, data controllers and lay representatives. Participants came from across the UK (England, Wales, Scotland and Northern Ireland) as well as Australia, Canada and the Netherlands.

After two short presentations summarising work carried out by the Public Engagement and Information Governance work streams of SHIP, workshop participants took part in small group discussions^1^ which focused on the following key questions:

- What does trust mean to you?
- What do you think makes a researcher trustworthy?
- Do you think enhancing trust (or procedures for enhancing trust) hinder or enable researchers in any way?

The discussions were facilitated, recorded and lasted around 35 minutes, after which time key findings from each of the groups were fed back to the whole group, and closing reflections were offered.

This paper presents findings from the focus groups with members of the public and from the stakeholder discussions at the workshop in order to illustrate the various ways in which trust and trustworthiness were understood in relation to SHIP and to research more broadly.

Throughout the paper the different parts of this research project are referred to as ‘focus groups’ and ‘stakeholder workshop’. Using the term ‘stakeholder’ to refer to the participants of the ‘stakeholder workshop’ is not intended to suggest that focus group participants are not also stakeholders. Clearly, as data subjects and potential beneficiaries of data-linkage research, focus group participants are also stakeholders in SHIP. There are also some overlapping interests between participants in the stakeholder workshop and the focus groups as the workshop also involved lay representatives. Inevitably, regardless of their professional roles, all participants are also members of the public and data subjects. As such, although the stakeholder workshop enabled exploration of professional and informed perspectives, the distinction between the characteristics and interests of workshop and focus group participants is not altogether clear-cut. However, for sake of clarity in discussing the two components of the research the terms ‘focus group participants’ and ‘stakeholders’ are used throughout the paper.

The analysis of discussions from the focus groups and stakeholder workshop followed an inductive approach to identifying themes within and across the discussions. This aimed to identify areas of agreement among the participants but also to highlight the diversity of views, interests and concerns which were expressed. Accordingly, the following discussion engages with the range of attitudes and responses articulated, and does not seek to make generalised statements about public opinion or preferences.

## 4. Findings

Across the focus groups and the discussions at the stakeholder workshop it was evident that trust was a highly salient concept. As will be illustrated below, judgements of actors’ or institutions’ trustworthiness were often central to focus group participants’ responses and attitudes. In particular, there were sharp contrasts between participants who trusted that research would operate in ‘the public interest’ and those who were generally more sceptical of the intentions and interests of researchers or research institutions. Such judgements of trustworthiness strongly influenced the extent to which individuals supported the aims of SHIP. For example, some focus group participants were generally supportive of SHIP since they trusted that data would be used for appropriate and necessary purposes, and that research would (at least probably) ultimately lead to benefits for healthcare:

I’ve got a very simple and naive answer for this. And it’s this. The state knows most things about you anyhow. So if … the more information the state has and the apparatus of state, the better they can handle the people and make … from a medical point of view, it’s to make them better. I know that sounds terribly naive, there are lots of other issues that probably people will bring up. But that’s the way I see it. The more information they get about the populous, the better it will be for them in the long run, and that’s the way I see it. (Mental Health Support Group1 – Male4)

Conversely, more sceptical focus group participants questioned whether research would necessarily or straightforwardly translate into benefits for healthcare. Moreover, some participants questioned the underlying justification of SHIP. For example, one participant suggested that there may be a hidden agenda:

My concern is that, I think at the end of the day this will in no way benefit any individual or any patient, I think there is a bigger agenda somewhere and I think having access to the kind of information that they are seeking to find is quite frightening and I’m not happy at all with any of it either, because what’s it for, what is it *really* for? (Black and Ethnic Minorities Group – Female6)

Other focus group participants expressed more ambivalent positions. For example, the following quote illustrates one participant’s ‘as-if’ trust (Wynne 1992, 1996, 2006) in that she acknowledges that data could be used for good or bad purposes but chooses to ‘put her belief in the system’:

… I suppose it’s back to that whole thing about using it for the power of good rather than the power of evil, isn’t it? […] I suppose […] you put your belief in the system that universities are there to try and sort of safeguard that this will be used for the correct reason. There’s all these things in place. And if we can use the information to benefit people, however that is, whether it’s their social care, their healthcare, their living circumstance, their longevity, then we would all be saying we see it as a good thing. But we always have that kind of wee devil on the other shoulder saying … (Mental Health Support Group1 – Female3)

Within the stakeholder workshop at the SHIP conference participants demonstrated a great deal of enthusiasm to engage on the topic of trust in research and highlighted that this was perceived as relevant in a number of ways. It was noted that there is no universal understanding of trust, and no way of ensuring that a project, activity or institution will be considered trustworthy by all parties since (as was evident in the focus groups) some people are likely to be more suspicious whilst others will be more trusting. However, workshop participants were widely agreed that trust is crucially important to research processes and institutions, and that if this is lacking it ‘could derail what we [as researchers] are doing’. There was therefore widespread acknowledgement that public trust was necessary and important for research.

### 4.1 Trust in who?

The focus group discussions indicated clear patterns in who was generally trusted by participants—or more specifically who was trusted to handle or manage individuals’ personal medical data. When asked who they felt should be responsible for the management of such data the majority of participants initially responded that this should be an individual’s healthcare provider (typically their GP). Participants routinely expressed high levels of confidence in healthcare providers’ competence at handling personal data appropriately and sensitively. For example, it was widely asserted that medical records should be shared between health practitioners and many participants noted that they were happy for their information to be shared within the NHS. However, typically they were not sure how extensive data sharing currently was, or which parts of the NHS would have access to what information, and some difficulty was experienced in trying to define for whom access to data would be relevant and/or necessary.

… There’s obviously thousands of people working in the NHS, at what point do you say, “Well, you can have access to it, but you can’t.” Is it people who’ve got daily contact with patients, is it researchers, is it consultants and doctors, paramedics, how do you define who’s going to get access or not? (Youth Group – Female5)

Although there were a few exceptions, in general participants were happy for their medical records to be shared with the doctors and specialists involved in their healthcare. However, most participants (at least initially) contended that information from their records should not be shared with anyone who is not directly involved in their healthcare. Varying levels of trust were expressed in non-clinical NHS employees. In particular, concerns were frequently raised about receptionists having access to medical records and confidential patient information. For example, there were concerns that receptionists might misuse information or look up the records of people they know. Nevertheless, for many focus group participants the NHS, as an institution, was highly trusted and many participants stated that they were content for data-management and data-sharing processes to be governed from within the NHS. In particular, several participants recounted that NHS computers have high levels of security and that members of NHS staff must abide by strict codes of conduct. This demonstrated a certain level of confidence in the NHS to oversee how data is protected. However, there was some concern about data being passed outside of the NHS:

But then how well are the people, I mean there’s quite a lot of screening goes on to the employment of people in the NHS, you know, to professional people and does the same standards apply to other agencies that would have access to your records. (Diabetes Support Group – Female6)

Nevertheless, it should be noted that many participants did not share this level of trust in the NHS. For example, participants acknowledged the potential for people within the NHS to misuse personal data:

What if someone got a hold of it, that’s what I think […] But by someone in the NHS for example being unprofessional, getting a hold of anyone’s information, I just think that’s a concern. Unless they were using it for a health purpose then fine, but it just feels that someone could just go on and, right, I know their name and date of birth, address, I can find out everything about them. (Youth Group – Female5)

A number of focus group participants demonstrated significantly different perceptions of primary healthcare providers (e.g. GPs) and other professionals within the NHS, or of the NHS as an institution. Given that many individuals have existing relationships with particular primary healthcare providers, in some cases built up over many years, and that these are the professionals in the NHS with whom individuals are likely to be most familiar, this may suggest that a familiar relationship with an identifiable individual is important for securing public trust. This reflects the observation that relationships of trust are, at least partly, based on emotional ties between individuals and affective judgements of the trustworthiness of individuals (Rowe and Calnan 2006).

The importance of relationships to trust was also a recurring theme throughout discussions at the stakeholder workshop. Stakeholders suggested that relationships of trust must be built up over time. Some workshop participants felt that it was easiest to build up relationships of trust when a research project had an individual in contact with research subjects. It was suggested that the human element of this was important and that trust could be facilitated through such things as being friendly, polite and considerate. By contrast, it was felt that where there is no individual relationship between members of a research team and research subjects, it can be more difficult to engender such trust. For research projects this level of familiarity between the research team and subjects may be easiest within a primary research context but may be more difficult to foster in secondary research.

### 4.2 Trust and altruism/commercialisation

Focus group participants who had been supportive of data sharing between healthcare professionals were typically more hesitant when asked to think about the ways in which personal data might be used for research. However, there was generally a preference for research to be conducted by academic researchers and participants frequently expressed high levels of trust in universities. For example, it was suggested that the involvement of universities gave participants greater confidence in the systems in place:

… you put your belief in the system that universities are there to try and sort of safeguard that this will be used for the correct reason. (Mental Health Support Group1 – Female3)

I think the very fact that [universities]’re involved in it speaks volumes for me. That helps me to accept it or otherwise these great places wouldn’t be involved. They’re institutions full of great academics. (Mental Health Support Group1 – Male4)

Of course, caution is needed here since the focus groups were being run by an academic researcher and this may have influenced participants’ responses. Yet, it should be noted that the participants were not asked directly how they felt about university or academic involvement in research, but rather raised this themselves. Through discussions it was clear that an important factor influencing positive perceptions of academic researchers was a perception that they were more altruistic than non-academic researchers. In particular, focus group participants often noted that they felt most comfortable with academic researchers as they were not expected to be motivated by profit.

Focus group participants were generally uncomfortable with the idea of organisations or private companies making profits out of access to personal medical data. In particular, there was concern that drug companies might exploit the NHS by using information from medical records to develop new drugs which they would then sell back to the NHS:

Because they’re the ones who make the profit in the end, because if they get all this information free and then they sell back the drugs to the NHS at exorbitant prices. (Diabetes Support Group – Female5)

Many focus group participants were concerned about the possibility of private companies (most notably pharmaceutical companies) having access to information in medical records or being involved in research which would access this information. This reflects a widely held concern that:

… an awareness of any profit motive underlying scientific research will eventually lead to significant erosion in trust, and a devaluing of science by the community. (Critchley 2008: 310)

It is frequently asserted that the public is less trusting of research which is conducted by private companies/organisations, and that the creation of profit from research is a key factor influencing this mistrust (Critchley 2008; Critchley and Turney 2004; Hargreaves et al. 2002). Previous research has highlighted that commercial involvement is an area of public concern (Grant et al. 2013; Hill et al. 2013; Nair et al. 2004). It has also often been assumed that members of the public have little or no understanding of the current role of commercialisation in research (Millstone and van Zwanenberg 2000; Van Gend 2002). Yet, qualitative and deliberative research which has engaged with this topic has found that while members of the public have concerns about the commercialisation of research they are often aware of its role and acknowledge the relevance of private company involvement in research (Davidson et al. 2013; Grant et al. 2013; Haddow et al. 2007). Moreover, Haddow et al. (2007) found that members of the public may be accepting of commercialisation so long as appropriate conditions are met (e.g. through benefit sharing). Similarly, while participants in our focus groups had concerns, in general they were not entirely opposed to commercialisation and often acknowledged the relevance of pharmaceutical company access/involvement in research. In line with the findings of Haddow et al. (2007), many focus group participants accepted that pharmaceutical companies had a role to play in public health research and would support this so long as there were sufficient safeguards in place to protect against inappropriate use of the data.

The ones who are, you know, of course for the purpose of advancement of science and treatment of the patients and diseases they should be involved, but how, again it comes to the mechanisms and the ways of controlling them, not any pharmaceutical company should have access, but those who are involved in the research, a particular research, they should certainly have access to certain information which are required by them, but again, that should be certainly safeguarded and controlled. (Black and Ethnic Minorities Group – Male4)

Confidentiality was considered to be of particular importance when records might be accessed by private companies such as pharmaceutical companies. In such instances anonymisation of data was generally viewed as being of greater importance than it otherwise would be. However, some focus group participants also questioned whether pharmaceutical companies would be interested in individuals. For example, in a focus group with a mental health support group several participants expressed doubt that drug companies would want individual-level information. It was suggested that in most cases they would only be interested in aggregate data or statistics and this was not viewed as a major concern. However, in several other focus groups there were significant concerns that identifiable information could be misused by private companies (such as pharmaceutical companies) for marketing purposes.

As such, although there was an evident pattern of higher levels of trust in academic researchers and healthcare professionals, and lower trust in private companies, these relationships were not straightforward or static. Rather, participants indicated that the extent to which they would trust particular researchers depended on a range of institutional factors and assurances about necessary and appropriate safeguards being in place.

### 4.3 Trust and transparency

A key theme to emerge through discussions at the stakeholder workshop was the connection between trust and transparency. Many workshop participants considered openness about research practices and outcomes to be crucial for ensuring public trust. For example, one workshop participant described a responsibility to inform data subjects of how their data was being used and to provide feedback on outcomes of this use. Most workshop participants agreed that public engagement should be focused on communicating positive messages about how data is used: ‘promoting the success stories’. As such, while it was noted that public engagement should not involve manipulating or ‘spinning’ information, it was felt that researchers or data controllers should be more proactive in communicating the positive aspects of research and data use. It was also suggested that there would be some benefit in raising public awareness of the complex legal environment surrounding data sharing and that this might demonstrate the legitimacy of researchers’ access to data. Similarly, it was contended that there is a lack of understanding of what researchers actually do, or of how they use data. One workshop participant suggested that most people imagine researchers to be based in laboratories and are not aware of types of research involving data analysis. It was said that members of the public have no experiential knowledge of this type of research and that this can lead to low understanding and lack of trust. Raising public awareness was therefore considered key to ensuring public trust. In these ways transparency and public engagement were largely viewed as opportunities for awareness raising or information provision (or even public relations).

Focus group participants also pointed to transparency as being important for ensuring public trust. In many cases focus group participants’ concerns or reservations about SHIP stemmed from a perceived lack of openness about the ways in which data are currently collected and used or how these processes are governed. For example, participants described a sense of inequity in that they felt that they were expected to allow more and more people to have access to their information but that they were not expected to want access to information about how it was being used, for what purposes or by whom:

This is what I feel they want to know all about us, but we’re, we’re not supposed to know all about them sort of thing whose doing it. (Diabetes Support Group – Female5)

Some focus group participants suggested that this lack of openness may be a deliberate effort to withhold information from the public and pointed to an awareness of previous instances where public information had been used and/or disseminated without public knowledge:

And Governments are also … our Government let’s not generalise too widely, our Government is not very good at transparency with things like data, at saying what it’s going to do, what it currently plans and then saying it changes its mind somewhere along the line. (Social Science Researchers – Female3)

In particular, and to a certain extent corroborating the discussion at the stakeholder workshop, focus group participants wanted greater information about how processes to manage requests for data access would be overseen and about who would be accountable for any breaches of privacy and/or misuse of data. One focus group participant (a nursing researcher) stated:

I do research, and my research is really important to me that I keep this data sensitive and there is no tracing to it. And it’s almost like who’s going to do that? You know, who’s going to look after this? Who’s going to ensure that there isn’t a breach of these aspects when they’re going to people who don’t maybe have such ethical governance. (Nursing and Midwifery Researchers – Female5)

As such there were calls for greater openness and transparency in relation to how data is currently used, and how requests for data access are managed:

It is important, I think the public should definitely be more informed and well informed and quite clearly explain to people why the data has been collected and what purpose and how it is used. I think they have a right to know. (Black and Ethnic Minorities Group – Male2)

However, in contrast to the position advanced in the stakeholder workshop, focus group participants emphasised that it was important that the information that was provided should be accurate, impartial and uncensored. Some focus group participants contended that any initiatives to raise awareness should be run or overseen by an independent body in order to avoid biased or inaccurate information:

I’d like to see an NGO definitely working on the, just constantly thinking about that education campaign and, you know, how that represents itself but somebody like a liberty, I mean, a civil liberty group involved, making sure, you know, because you can … the way you present something on a television advert in terms of, okay, we’re now doing this and it will help us cure cancer, or help us deal with this, oh by the way, we’ll … it will also make sure that, you know, people know exactly where you live! You know, but don’t worry about that, we’re curing cancer! You know, it would definitely need regulation in terms of how that gets presented. (Social Science Researchers – Male2)

The different interpretations of transparency demonstrated at the stakeholder workshop and within the focus groups illustrate differing understandings of the relationship between science and the public, and of the role of public engagement. While stakeholder workshop participants referred primarily to ‘informational transparency’ implying openness about how data are used and the value of data-linkage research, focus group participants were largely more concerned about ‘participatory transparency’ and ‘accountability transparency’ calling for openness about governance and decision-making practices (Brown and Michael 2002). In this way, much of the discussion at the stakeholder workshop could be viewed as exemplifying a deficit model of public engagement, whereby public trust can be ‘improved’ through the provision of appropriate (and selective) information. Conversely, focus group participants indicated that they would appreciate a more open exchange of information and greater equity in the science–public relationship. Whilst stakeholders at the workshop discussed public engagement as a means of generating public trust in research/researchers, focus group participants viewed public engagement as a potential indicator of the trustworthiness of the research and/or researchers.

### 4.4 Trust and trustworthiness

The emphasis on trustworthiness of research/researchers as opposed to public trust in research/researchers is an important theme which emerged from the focus groups. The extent to which focus group participants considered SHIP to be trustworthy strongly influenced their responses. In particular, reflecting the emphasis on ‘accountability transparency’ noted above, this led to calls for more information about how SHIP would operate. During the focus groups participants asked many questions about the ways by which processes within SHIP would be governed and how access to personal medical information would be controlled at an institutional level. For example, it was asked:

I wonder who the captain of this ship is really then? You know, like the gatekeeper? (Nursing and Midwifery Researchers – Female5)

Similarly, it was stated:

Also I think there’s always a danger of leakage as well, I think it can get everywhere, I think you need to be aware of that as well whether the health services control or whether pharmaceutical research companies, etc. I think would be the main thing, who controls it, who is responsible for it, and how much information is out there or how much information they can access. (Black and Ethnic Minorities Group – Female3)

Focus group participants acknowledged that as individuals they had little control over how data-sharing processes were governed:

It’s also a bit like pension funds in the sense that it’s a big complicated set up that as one person, we don’t really have much control over what happens […] So, I might like my pension fund […] not to invest in the arms industry, for example, or tobacco industry, but it’s actually quite hard to change something that big as one person. (Social Science Researcher – Female3)

As such, who is in control of these processes and decisions was an important consideration influencing focus group participants’ attitudes and responses, and this was one area about which participants indicated they would like further information. The focus group participants also suggested that members of the public should have a role in overseeing processes within SHIP and that lay representatives could play an important role in ensuring accountability and the protection of public interests.

However, regardless of who is in control of the processes and mechanisms governing access to medical records data, the majority of focus group participants contended that misuse of data or breaches of privacy would inevitably occur from time to time. As such an important consideration related to accountability and what would happen in instances of misuse of data. One participant noted:

Do you think that perhaps the reason we’re not happy with many people having that level of power over our data, partly because we don’t believe that the penalties for misusing data are severe enough. I mean, for me, that’s a crucial point, I actually think there would be less mismanagement of data if the penalties of knowingly selling or giving away personalised information carry far greater criminal penalties [… Currently] They don’t, I mean, you’re not going to go to jail for it! Whereas, perhaps if you did people would be less likely to, you know, purposely sell personalised information. (Social Science Researchers – Male1)

Moreover, it was contended that there may be powerful interests preventing such cases resulting in penalties or prosecution:

Its not only the penalties you have to recognise that the people, the companies that are going to be using the information are going to have an awful lot of money so, like, there’s the question of whether you even got as far as a penalty. (Social Science Researchers – Male3)

Make it a whacking big penalty. (Social Science Researchers – Female3)

No, the way you … you’re looking for safeguards and someone, like, this will actually be applied fairly and then you’ve got your distrust of some legal system and various biases within it and, sort of, individual versus corporate power. You tend to assume that the corporate is going to win. (Social Science Researchers – Male3)

Participants in many of the focus groups demonstrated scepticism about the existing governance mechanisms or oversight procedures. There was some concern that committees of oversight bodies would operate with a presumption in favour of allowing data sharing for research to go ahead and that a range of commercial or political interests may have influence in preventing or impeding robust accountability procedures.

Participants at the stakeholder workshop also stated that breaches were inevitable and suggested that public trust was important for avoiding negative responses to such breaches. Simultaneously, stakeholders also contended that how researchers or institutions respond to breaches is important as such responses can either foster or damage public trust, again emphasising the importance of ‘accountability transparency’.

Stakeholders at the workshop expressed varying, and at times conflicting, views on the role of governance mechanisms in relation to trust in research and/or researchers. For some, governance systems were crucial for ensuring and maintaining trust. However, for others the existence of complex governance mechanisms and safeguards was itself potentially a source of mistrust in researchers or research institutions. For example, one workshop participant suggested that members of the public might respond to governance mechanisms/systems by asking: ‘Why does research require all this? What are you trying to protect us from?’. As such there was concern that an awareness of governance mechanisms could lead to suspicion. Nevertheless, for many workshop participants compliance with standards set through governance systems was considered crucial for ensuring trust in research and/or researchers. It was argued that people trust researchers because they assume that there are oversight and governance processes in place and that researchers will comply with these. Compliance was therefore viewed as crucial for trust, however, one workshop participant commented that ‘whether compliance is sufficient is another question’.

## 5. Discussion and conclusions

Public trust in science continues to be a topic of much academic and policy debate. In our research it has been very clear that trust is also perceived as a salient issue for both researchers and members of the public. However, reflecting recent work in the science and technology studies literature, it is clear that the public’s relationships of trust and/or mistrust in science and research are not straightforward. Such relationships have been shown to be characterised by ambivalence and public trust has been demonstrated to be highly conditional and variable. Thus, this paper has aimed to move beyond simple descriptions of whether publics trust researchers, or in whom members of the public place their trust, and to explore more fully the bases of public trust/mistrust in science, what trust implies and equally what it means for research and/or researchers to be trustworthy. The research methods also represent an example of increasingly frequent public engagement activities associated with large science projects which—it should not be denied—are themselves an effort to increase public trust and to ensure RRI.

Within the focus groups there were clear patterns in which actors were generally considered to be more or less trustworthy (i.e. primary healthcare providers and academic researchers were generally considered more trustworthy than commercial actors such as pharmaceutical companies). However, this pattern did not straightforwardly translate into support for research conducted by healthcare professionals or academics and opposition to research conducted by pharmaceutical companies. Focus group participants demonstrated an awareness of the realities and practicalities of research and, in particular, noted that it may be relevant or necessary for pharmaceutical companies to be involved in or conduct research using personal medical data. Equally, participants’ generally higher levels of trust in academic researchers did not mean that they were happy for academic researchers to have unfettered access to personal medical data. Rather, participants’ responses and their levels of support for data sharing and researcher access to personal medical information depended on a range of factors such as: institutional arrangements for data-sharing processes, transparency of processes and the existence of robust accountability procedures. The extent to which individuals perceived research institutions or data controllers—whether public or private, academic or commercial—to be transparent and to ensure high levels of accountability was crucial to informing their responses. Moreover, the extent to which individuals anticipated that members of the public could have control over their personal medical data, or could play a role in overseeing data-sharing processes also influenced perceptions of trustworthiness.

Members of the SHIP SMG emphasised that public engagement should aim to foster high levels of public trust in SHIP. As noted above, for some members of the SMG this trust was expected to come about through information provision and awareness raising. Similarly, stakeholders within the workshop at the SHIP conference suggested a need for greater transparency and public engagement in order to ensure public trust in research. For many workshop participants this transparency should focus on communicating positive messages about the value, importance and benefits of data sharing for health research. However, although awareness raising has a role to play, the focus groups have demonstrated that transparency must go much further than the selective communication of positive messages if it is to secure public trust. Instead, public participants in the focus groups emphasised the importance of trustworthiness within research and data-sharing processes. Transparency may be one indicator of trustworthiness, but requires open communication of uncensored information. Therefore, research/researchers will be more likely to be perceived as trustworthy if transparency and public engagement involve open dialogue with members of the public and opportunities for deliberation, rather than controlled dissemination of information.

This emphasis on transparency reflects broader attention to this area over recent years. There has been increasing emphasis on transparency as a mechanism for addressing lack of public trust in science and scientific institutions. As Brown and Michael (2002: 260) have noted:

… in seeking to resolve the problems of trust and credibility, transparency has become ever more central to the revalidation of otherwise increasingly circumspect professions, institutions and commercial organisations.

However, this emphasis on transparency can conceal its problematic nature. For example, as illustrated in the various ways that the stakeholder workshop and focus group participants discussed transparency, this can be pursued and achieved in different ways. Transparency might take the form of: informational transparency requiring disclosure of information on which decisions are based; participatory transparency, enabling public participation in decision-making processes or; accountability transparency whereby decision-makers are held accoun*table* (see Balkin (1999), discussed in Brown and Michael 2002). Moreover, as noted by Brown and Michael (2002), ensuring transparency does not represent a simple solution to low levels of public trust since trust may itself be a necessary precondition for transparency being perceived as adequate or genuine. Low levels of public trust lead to public scepticism of participatory or consultative events and of the individuals or organisations facilitating them. Where trust is not already present participants or observers are likely to be sceptical of the level of transparency enacted. Therefore, transparency alone may be inadequate to build trust, instead a circular conundrum emerges whereby transparency is necessary to build trust, but trust is required in order for the transparency to be recognised as adequate (Brown and Michael 2002). Brown and Michael (2002) argue that in order to break this circle what is needed is not more openness but rather more ‘authenticity’. This authenticity this can be signalled through emotional engagement and demonstration of the pain or suffering endured through decision-making processes. They contend that demonstrations that decision-makers have attempted to engage, incorporate and address disparate views to such an extent that it has caused them distress or suffering give them authenticity which, in turn, builds trust and lends confidence in the transparency of decision-making.

This has important implications for public engagement in interdisciplinary projects. While public engagement is routinely conceptualised as a mechanism for ensuring public trust, such approaches may be of limited value. Public engagement can more appropriately be viewed as a mechanism for ensuring the trustworthiness of research. Yet such exercises are also performances of authenticity—that is they represent attempts to demonstrate to wider publics that institutions or programmes such as SHIP are meaningfully grappling with the challenges of addressing disparate viewpoints. Such public engagement exercises can have many benefits: providing insights into how research, or researchers are perceived; what concerns or preferences exist and to what extent practices and aims reflect public values; providing opportunities for researchers to reflexively address their own trustworthiness and seek to build high levels of trustworthiness into research practices and institutional arrangements. However, as Brown and Michael (2002) argue, such processes may not be adequate to build trust where this is not already present (at least in low levels). As such public engagement exercises can aim to build relationships with publics to engender trust through demonstrating personal and emotional commitments to transparency and participation. While there is no simple toolkit for building public trust such open, human processes are likely to be more fruitful means of ensuring sustainable public trust compared to more traditional approaches to awareness raising or consultation.

However, that is not to deny an important role for awareness raising and the provision of information. Within the focus groups there were clear examples of areas about which members of the public would like more information (i.e. how is personal medical data currently used and what safeguards are in place to protect confidentiality), but this information provision is likely to be most effective when it responds to public questions or concerns rather than pre-emptively selecting what the public should (and should not) know. As such public engagement is likely to be most effective when it incorporates dialogic and deliberative forms of communication. Thus, efforts to improve transparency should be focused on ‘informational transparency’, ‘participatory transparency’ and ‘accountability transparency’ simultaneously. In such a way it is not simply an opportunity for publics to learn about science or research, but also for scientists or researchers to learn about the ways in which their work is perceived by, and impacts on, publics and to what extent it reflects public values. Thus, public engagement should not be aimed at ‘improving’ public trust in science, but rather at improving the trustworthiness of science.

As the international interest in secondary uses of routinely collected data grows this becomes an ever more salient topic for research institutions, funders and governments. In the light of recent controversies surrounding care.data in England and NEHRS in Australia (to give but two examples), if the ambitious plans for ‘optimising data sharing for research’ in order to ‘derive maximal societal benefit’ (Medical Research Council and Wellcome Trust 2006) are to be realised ensuring that such programmes have public support and are widely viewed to be operating in the public interest will be essential. This necessitates considerable attention being paid to ensuring that such programmes are regarded as trustworthy by members of the public and requires significant efforts to not only maximise transparency in data sharing and governance processes, but also to build and maintain relationships with wider publics to foster trust in open and meaningful ways.

## Footnotes

1. These small group discussions followed a similar format to the focus groups in that they were semi-structured group discussions. However, in order to avoid confusion in the discussion throughout the paper a distinction is made between the focus groups with members of the public and small group discussions which formed part of the stakeholder workshop.
